# A “Valve‐Closing” Starvation Strategy for Amplification of Tumor‐Specific Chemotherapy

**DOI:** 10.1002/advs.202104671

**Published:** 2022-01-17

**Authors:** Xianglong Li, Cong Jiang, Qinghua Wang, Shaobo Yang, Yuanyuan Cao, Ji‐Na Hao, Dechao Niu, Yan Chen, Bo Han, Xin Jia, Peng Zhang, Yongsheng Li

**Affiliations:** ^1^ Lab of Low‐Dimensional Materials Chemistry Key Laboratory for Ultrafine Materials of Ministry of Education Frontier Science Center of the Materials Biology and Dynamic Chemistry Shanghai Engineering Research Center of Hierarchical Nanomaterials School of Materials Science and Engineering East China University of Science and Technology Shanghai 200237 P. R. China; ^2^ Department of Thoracic Surgery Shanghai Pulmonary Hospital Tongji University School of Medicine Shanghai 200092 P. R. China; ^3^ Key Laboratory of Xinjiang Endemic Phytomedicine Resources of Ministry of Education School of Pharmacy Shihezi University Shihezi 832003 P. R. China; ^4^ Key Laboratory for Green Processing of Chemical Engineering of Xinjiang Bingtuan School of Chemistry and Chemical Engineering Shihezi University Shihezi 832003 P. R. China

**Keywords:** differential stress sensitization, glucose metabolism, GLUT1 inhibition, stability‐controllable nanomedicines, starvation‐sensitized chemotherapy

## Abstract

Starvation‐dependent differential stress sensitization effect between normal and tumor cells provides a potentially promising strategy to amplify chemotherapy effects and reduce side effects. However, the conventional starvation approaches such as glucose oxidase (Gox)‐induced glucose depletion and nanomedicine‐enabled vascular embolism usually suffer from aggravated tumor hypoxia, systemic toxicity, and unpredictable metabolic syndrome. Herein, a novel “valve‐closing” starvation strategy is developed to amplify the chemotherapy effects via closing the “valve” of glucose transported into tumor cells, which is accomplished by a glucose transporters 1 (GLUT1, valve of glucose uptake) inhibitor (Genistein, Gen) and chemotherapeutic agent (Curcumin, Cur) coloaded hybrid organosilica‐micelles nanomedicine (designated as (Gen + Cur)@FOS) with controllable stability. In vitro and in vivo results demonstrate that (Gen + Cur)@FOS can effectively reduce glucose/adenosine triphosphate levels in tumor cells by inhibiting GLUT1 expression (i.e., “valve‐closing”) to induce the starvation of tumor cells, thus weakening the resistance of tumor cells to apoptosis caused by chemotherapy, and consequently contributing to the remarkably improved antitumor efficiency and minimized side effects based on the stress sensitization effect mediated by GLUT1 inhibition‐induced starvation. This “valve‐closing” starvation strategy provides a promising paradigm for the development of novel nanotherapeutics with amplified chemotherapy effect.

## Graphical abstract

A novel “valve‐closing” starvation strategy to amplify the chemotherapy effect is developed based on GSH‐responsive organosilica hybrid micelles encapsulated with glucose transporters 1 inhibitor genistein (Gen) and drug curcumin (Cur), in which Gen can effectively starve tumor cells to enhance their sensitivity to Cur and minimize side effects of chemotherapy by starvation‐mediated stress sensitization.

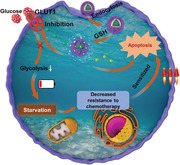

## 1 Introduction

With the booming development of nanomedicine, various oncology therapy strategies have been developed to fight cancers.^[^ ^1^ ^]^ Among them, chemotherapy is one of the most important cancer therapy modalities, but its severe side effects impose great clinical challenges.^[^ ^2^ ^]^ Although different nanocarriers have been exploited to improve the efficiency of chemotherapeutic drug delivery and reduce the side effects, the unique metabolic changes of tumor cells cause their resistance to chemotherapeutic interventions.^[^ ^3^ ^]^ Typically, chemotherapeutic drugs achieve their treatment outcome by causing apoptosis, i.e., disrupting the intracellular balance between prosurvival and proapoptosis. However, evasion of apoptosis is an instinctively self‐protective behavior of tumor cells, resulting in low antitumor efficacy. Recently, studies have demonstrated that short‐term fasting or fasting‐mimicking diets can make cancer cells more sensitive to chemotherapy, while protecting normal cells against the toxic effects of chemotherapy drugs, which is termed as differential stress sensitization.^[^ ^4^ ^]^ Specifically, in the starvation state, normal cells tend to switch themselves from the normal proliferative state toward a maintenance state to save sufficient energy for sustaining their organelle's function to protect themselves against the toxicity of antitumor drugs. However, because of the presence of oncogenic mutations, tumor cells are always in a high metabolic and proliferative state regardless of the energy shortage. The insufficient energy supply will cause cancer cells’ organelle dysfunction, resulting in their inefficient responses to external stress including chemotherapy and thereby rendering them more vulnerable to chemotherapy.^[^ ^5^ ^]^ Based on this differential stress sensitization effect, fasting‐assisted chemotherapy represents a promising way to improve the efficacy of chemotherapy while reducing the toxic effects on normal tissues.^[^ ^5^ ^]^ However, fasting is hardly tolerated by patients due to nutrient absence and possibly causes metabolic syndrome. Therefore, developing a nondietary starvation approach is highly desired to improve chemotherapy efficiency by minimizing the resistance of tumor cells to apoptosis induced by chemotherapeutic agents.

Compared to normal tissues, tumor cells require a much higher amount of glucose uptake and undergo faster glucose metabolism through anaerobic glycolysis to survive and proliferate, a phenomenon known as the “Warburg effect.”^[^ ^6^ ^]^ Based on this metabolic difference, two main approaches to starving tumors have been developed by glucose depletion and vascular embolism.^[^ ^7^ ^]^ Typically, glucose oxidase (GOx)‐induced starvation that can efficiently consume glucose in tumor cells has received increasing attention.^[^ ^8^ ^]^ However, the poor stability and systemic toxicity of GOx greatly limit the development of such GOx‐based nanomaterials.^[^ ^9^ ^]^ Besides, the GOx‐catalyzed reaction also depletes oxygen in solid tumors and generates the H_2_O_2_ byproducts, thus accelerating tumor invasion and metastasis, and causing irreversible oxidative damage.^[^ ^10^ ^]^ Vascular embolism is another cause tumor starvation strategy by blocking the blood flow and nutrition supply. However, rapid blood flow flushing impedes the formation of agglomerate or delivers it to other organs, rendering vascular occlusion ineffective or causing unsuspected thrombosis. Therefore, it is highly imperative to exploit a new starvation strategy to overcome these challenges.

**Scheme 1:**
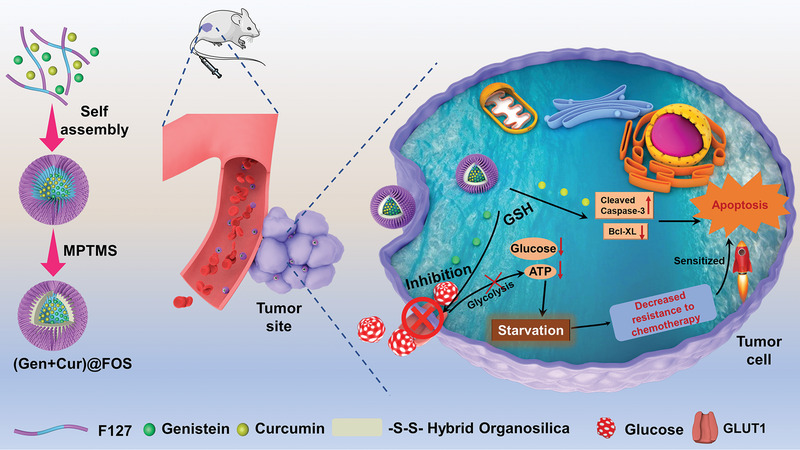
Schematic illustration for the synthesis of (Gen + Cur)@FOS with GSH‐responsive activity for GLUT1‐inhibited starvation‐sensitized tumor‐specific chemotherapy.

Glucose transporters (GLUTs), as an important transmembrane protein family, regulate the entry of extracellular glucose into cells and serve as the “valve” to control the constant glucose uptake of cells.^[^ ^11^ ^]^ Due to the “Warburg effect,” elevated expression levels of GLUTs have been observed in tumor cells to meet their requirements for enhanced glucose supply during the fast‐growing of tumors.^[^ ^12^ ^]^ GLUT1, whose expression level is much higher than that of the other 14 human GLUTs family members, is considered as the main prognostic indicator for tumorigenesis. Nevertheless, few studies on exploring the starvation strategy by suppressing GLUT1 have been reported so far, though the GLUT1‐targeting nanomedicines have been developed to mediate their endocytosis.^[^ ^13^ ^]^ Herein, we develop a novel “valve‐closing” starvation protocol to enhance the chemotherapy effects by encapsulating genistein (Gen) as the GLUT1 inhibitor and curcumin (Cur) as the chemotherapeutic agent into a stability‐controllable nanocarrier based on the newly fabricated organosilica hybrid micelles, named as (Gen + Cur)@FOS (**Scheme** **1**). With the accumulation of (Gen + Cur)@FOS at the tumor sites, the overexpressed glutathione (GSH) induces the biodegradation of the hybrid organosilica containing disulfide bonds, resulting in the release of the loaded cargoes. Both in vitro and in vivo results demonstrate that the released Gen from (Gen + Cur)@FOS can specifically inhibit the expression of GLUT1 to block the glucose uptake pathway of tumor cells (i.e., “valve‐closing”), causing the starvation of tumor cells. Under such starvation state, the resistance of tumor cells to apoptosis induced by Cur is weakened, and thereby the chemotherapeutic effect of Cur is significantly amplified. Consequently, (Gen + Cur)@FOS exhibits excellent tumor therapeutic efficiency and minimal toxicity to normal cells/tissues through the starvation‐induced differential stress sensitization effect. This “valve‐closing” starvation strategy for amplification of tumor‐specific chemotherapy provides an instructive design for the treatment regimens.

## 2 Results and Discussion

**Figure 1:**
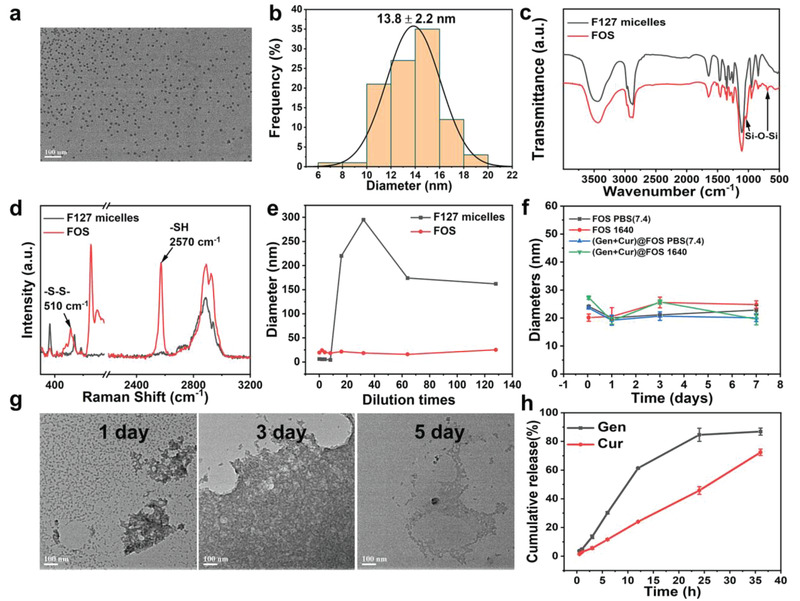
a) TEM image of FOS (scale bar: 100 nm). b) Particle size statistics of FOS based on (a). c) FT‐IR spectra of F127 micelles and FOS. d) Raman spectra of F127 micelles and FOS. e) Dilution stabilities of F127 micelles and FOS tested by DLS measurement (initial concentration: 1 mg mL^−1^). f) Stabilities of FOS and (Gen + Cur)@FOS in different media (PBS and RPMI‐1640) for 7 days. g) TEM images of FOS incubated in PBS (pH = 7.4) with GSH (10 × 10^−3^ m) for different periods (scale bar: 100 nm). h) Cumulative drug release curves of (Gen + Cur)@FOS in PBS (pH = 7.4) containing 10 × 10^−3^ m of GSH.

The fabrication process of (Gen + Cur)@FOS is illustrated in Scheme 1. First, the hydrophobic Gen and Cur were simultaneously encapsulated into the cores of the micelles during the self‐assembly process of Pluronic F127 block copolymer approved by the U.S. Food and Drug Administration (FDA). Then, to endow the micelles with controllable stability (i.e., circulation biostability and stimuli‐responsive biodegradability), an inorganic–organic hybrid network containing disulfide bonds was subsequently formed by the hydrolysis/condensation of (3‐mercaptopropyl)trimethoxysilane (MPTMS) in the micellar cores, and the hybrid organosilica‐micelles nanocarrier (designated as FOS) loaded with Gen and Cur was obtained. Transmission electron microscopy (TEM) images indicate that the as‐synthesized FOS nanocarrier possesses high monodispersity and spherical morphology with a particle size of 13.8 ± 2.2 nm (**Figure** **1**a,b). Both Fourier transform infrared (FT‐IR) spectra (Figure 1c) and Raman spectra (Figure 1d) exhibit the appearance of characteristic peaks of Si—O—Si, —S—S—, and —SH groups in FOS, confirming the successful formation of disulfide bond‐doped hybrid cores in the F127 micelles, where the —S—S— bonds originate from the oxidation of partial —SH groups of MPTMS by O_2_ in the air during the hydrolysis/condensation reaction in an open system under a basic condition.^[^ ^14^ ^]^ Due to the abundant silicone hydroxyl and sulfhydryl groups on the surface, the FOS nanocarrier presents a more negative zeta potential than that of F127 micelles (Figure S1, Supporting Information), which facilitates the long circulation in blood and delivery efficiency through enhanced permeability and retention (EPR) effect.^[^ ^15^ ^]^

To verify the stabilities of the FOS against dilution, the concentration‐dependent hydrodynamic particle size changes of F127 micelles and FOS were compared. As shown in Figure 1e, the particle size of F127 micelles without cross‐linked core increased abruptly once the dilution times are increase over a critical point of 16, while that of FOS remains constant during the dilution treatment, even with a 128‐fold dilution. These suggest that the hybrid core can effectively protect the structure of F127 micelles from disintegrating below its critical micelle concentration, and enables the nanocarrier stable enough to prevent the loaded cargoes from leaking in the blood circulation. Moreover, the loadings of Gen and Cur during the self‐assembly process have no influence on the morphology, particle size, and zeta potential of the FOS nanocarrier, as demonstrated in Figures S2–S6 of the Supporting Information. Both the FOS and (Gen + Cur)@FOS show negligible changes in the particle diameters even after 7 days storage in both phosphate‐buffered saline (PBS) and RPMI‐1640 media (Figure 1f), confirming their relatively high biostabilities. To investigate the redox‐responsive degradation behavior of FOS, its morphological changes in PBS (pH = 7.4) containing 10 × 10^−3^ m GSH for different incubation periods were monitored by TEM (Figure 1g). It is observed that GSH triggered a significant morphological transition of FOS from monodispersion to agglomeration, followed by structural collapsing after incubation of 5 days (Figure 1g), proving that the disulfide bonds‐doped organosilica core in FOS enables it to responsively degrade and release cargoes via the GSH‐induced breakage of disulfide bonds. Following this, the GSH‐responsive drug‐release properties of (Gen + Cur)@FOS were measured. As shown in Figure 1h and Figure S7 (Supporting Information), up to 86.8% and 72.4% of Gen and Cur were released from FOS under GSH stimulus (10 × 10^−3^ m) within 36 h, respectively, which is much higher than that without GSH stimulus and accompanied by the color change of the simulation fluid from colorless to Cur's yellow (Figure S7B, Supporting Information). These suggest that the as‐synthesized FOS nanocarrier possesses controllable stability to guarantee the loaded cargoes’ safety in blood circulation and specific release in tumor tissues.

**Figure 2:**
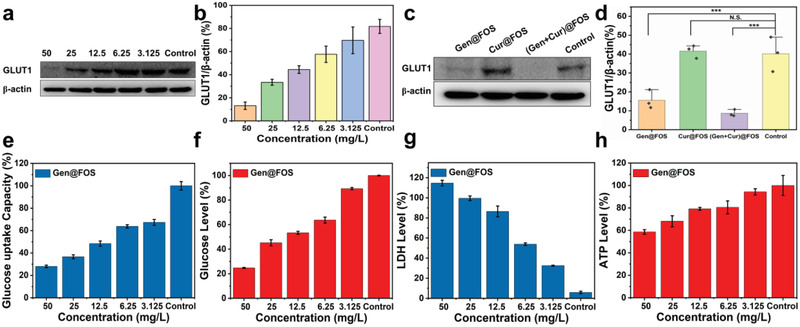
a) The expression of GLUT1 of HeLa cells before and after incubated with different concentrations (3.125, 6.25, 12.5, 25, and 50 mg L^−1^, [Gen]:[Cur] = 1:1) of (Gen + Cur)@FOS for 48 h. b) Quantification for GLUT1 protein expression from (a). c,d) The expression of GLUT1 of HeLa cells before and after treated with different nanomedicines of Gen@FOS, Cur@FOS, and (Gen + Cur)@FOS (50 mg L^−1^, [Gen]:[Cur] = 1:1), and the corresponding quantification analysis. e–h) The glucose uptake capacity, the glucose level, the LDH level, and the ATP level of HeLa cells after incubated with different concentrations of Gen@FOS ([Gen] = 3.125, 6.25, 12.5, 25, and 50 mg L^−1^) for 48 h, respectively. Statistical significances were calculated via Student's *t*‐test. *P < 0.05, **P < 0.01, and ***P < 0.001.

To validate the feasibility of GLUT1 inhibition‐induced starvation by the nanomedicine, the expressions of GLUT1 protein in HeLa cells incubated with different concentrations of (Gen + Cur)@FOS were tested by western blot. As shown in **Figure** **2**a,b, the GLUT1 expression (normalized to *β*‐actin) in HeLa cells is notably decreased with the increasing of (Gen + Cur)@FOS, and a 6.23‐fold inhibition of GLUT1 level is observed in HeLa cells after incubated with 50 mg L^−1^ of (Gen + Cur)@FOS. This reveals that (Gen + Cur)@FOS can effectively suppress the expression of GLUT1 and thus block the glucose uptake of HeLa cells from the extracellular matrix. To clarify the functional component of (Gen + Cur)@FOS for GLUT1 inhibition, the expression and quantification analysis of GLUT1 in HeLa cells treated with Gen@FOS, Cur@FOS, and (Gen + Cur)@FOS (50 mg L^−1^) were investigated, respectively. As shown in Figure 2c,d, the introduction of Cur@FOS without Gen loading has no effects on the GLUT1 expression in HeLa cells, while for the Gen‐loaded nanomedicines‐incubated HeLa cells, their GLUT1 expressions were significantly reduced. Meanwhile, the glucose uptake capacity and intracellular glucose level of HeLa cells exhibit a dose‐dependent decrease behavior and achieve a 3.57‐fold and 4.05‐fold reduction with 50 mg L^−1^ of Gen (Figure 2e,f), respectively, confirming that Gen serves as an effective GLUT1 inhibitor in the (Gen + Cur)@FOS to close the “valve” of HeLa cells’ glucose uptake and trigger their starvation. Such Gen‐induced GLUT1 inhibition for starvation was further verified by monitoring the content of adenosine triphosphate (ATP) and lactate dehydrogenase (LDH) in Gen@FOS‐treated HeLa cells, as tumor cells are forced to metabolize lactic acid under LDH catalysis after the glycolysis inhibition and thus cause the enhancement of LDH level.^[^ ^16^ ^]^ As anticipated, with the incubation concentration of Gen@FOS increasing, the LDH level in HeLa cells was significantly raised (Figure 2g), and up to 19.4‐fold enhancement was achieved at a high Gen@FOS concentration (50 mg L^−1^), suggesting that the Gen@FOS can effectively block the glucose‐based glycolysis process and thus induces the occurrence of lactate metabolic process in HeLa cells. Owing to this alternative lactate metabolic pathway of tumor cells, the reduction of intracellular ATP is slightly less efficient than that of GLUT1 level (Figure 2h), but the energy‐deprivation effect of tumor cells by Gen is still evident. These demonstrate that specific inhibition of GLUT1 expression by Gen‐loaded nanomedicine is an effective strategy to starve HeLa cells, which is expected to trigger the subsequent stress sensitization effect for amplifying the chemotherapy effect.^[^ ^5^, ^17^ ^]^

**Figure 3:**
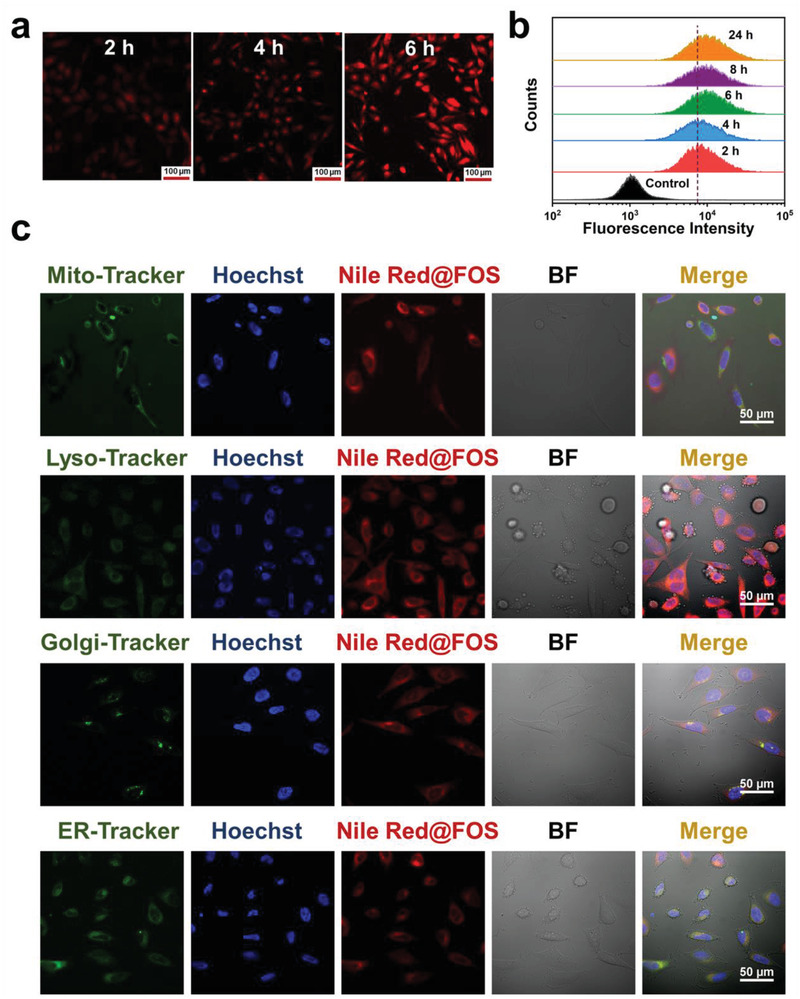
a) CLSM images of HeLa cells incubated with Nile Red@FOS for various periods (scale bar: 100 µm). b) Cell uptake of Nile Red@FOS with different incubation periods by flow cytometry analysis. c) High‐magnification CLSM images of HeLa cells incubated with Nile Red@FOS for 12 h. Mitochondria (Mito), lysosomes (Lyso), Golgi apparatus (Golgi), and endoplasmic reticulum (ER) were stained with the corresponding green fluorescent tracker. The nucleus was marked blue by the classic staining reagent Hoechst 33342 (Hoechst). The bright field is abbreviated as BF.

**Figure 4:**
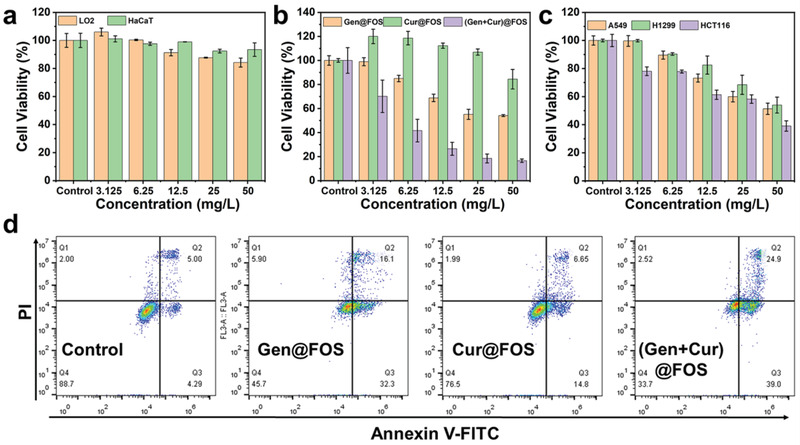
a) Cell viabilities of different normal cells treated by (Gen + Cur)@FOS for 48 h. b) Cell viabilities of HeLa cells treated by Gen@FOS, Cur@FOS, and (Gen + Cur)@FOS for 48 h. c) Cell viabilities of different tumor cells (A549, H1299, and HCT116) were incubated with (Gen + Cur)@FOS for 48 h. d) The apoptosis of Annexin V‐FITC/PI‐stained HeLa cells after treatments with Gen@FOS, Cur@FOS, and (Gen + Cur)@FOS for 48 h ([Gen] = 50 mg L^−1^, [Cur] = 50 mg L^−1^).

Inspired by the excellent (Gen + Cur)@FOS‐enabled starvation effects, the in vitro therapeutic efficacy of (Gen + Cur)@FOS to tumor cells was further evaluated. Initially, Nile Red was chosen as a fluorescent model to be encapsulated into the FOS nanocarrier to simulate the cell uptake behavior of (Gen + Cur)@FOS. Dynamic light scattering (DLS) result indicates that the particle size of Nile Red@FOS is identical to that of (Gen + Cur)@FOS (Figure S8, Supporting Information). Confocal laser scanning microscope (CLSM) images show that the red fluorescence of Nile Red@FOS‐incubated HeLa cells became brighter with time extending from 2 to 6 h, indicating that Nile Red@FOS was successfully endocytosed into the HeLa cells and accumulated in a time‐dependent manner (**Figure** **3**a). Further evidence was provided by flow cytometry analysis, which reveals that the fluorescence intensity of cytophagic nanomaterials increased with time extending and achieved uptake saturation at about 8 h (Figure 3b). To observe the location of FOS in the cellular suborganelles after endocytosis by HeLa cells, MitoTracker Green, LysoTracker Green, Golgi‐Tracker Green, ER‐Tracker Green, and Hoechst 33342 were employed to stain and visualize the mitochondria, lysosomes, Golgi apparatus, endoplasmic reticulum (ER), and nucleus of Nile Red@FOS‐treated HeLa cells, respectively. As shown in Figure 3c, more red fluorescence signals from Nile Red@FOS overlapped with the green fluorescence signals from ER‐Tracker Green stained ER after 12 h of incubation, indicating that FOS was mainly located in the ER. The cytotoxicity of (Gen + Cur)@FOS to different normal (human liver LO2 cells and immortalized keratinocytes HaCaT cells) and tumor cell lines was determined by cell counting kit‐8 assay. As shown in **Figure** **4**a, no significant changes of the normal cell lines’ viabilities are observed after (Gen + Cur)@FOS treatment even at a high dose of 50 mg L^−1^ for 48 h, proving the negligible toxicity of (Gen + Cur)@FOS to the normal cells. This can be ascribed to the low cargo release of (Gen + Cur)@FOS in normal cells as well as the protective effect of Gen‐induced starvation that mitigates the toxicity of Cur in normal cells.^[^ ^5^, ^17^ ^]^ As expected, the pure drugs of Gen and Cur exhibit obvious cytotoxicity to HeLa cells (Figure S9, Supporting Information). Figure 4b and Figure S10 (Supporting Information) show that the (Gen + Cur)@FOS displays remarkable concentration‐ and time‐dependent cytotoxicity against HeLa cells. Especially, with 48 h of incubation time, the cytotoxicity of (Gen + Cur)@FOS to HeLa cells is much higher than that of single Gen@FOS and Cur@FOS, verifying that the Gen‐enabled starvation amplifies the cytotoxicity of Cur to the tumor cells through enhancing their sensitivity toward the chemotherapy drugs. It is observed that the Cur@FOS has a slight promotion on cell proliferation at low concentrations, which can be attributed to the autophagic protection of tumor cells.^[^ ^18^ ^]^ Furthermore, the cytotoxicity of (Gen + Cur)@FOS to various tumor cell lines (A549, H1299, and HCT116) was also studied. As shown in Figure 4c, (Gen + Cur)@FOS also exhibits distinct cytotoxicity against the three different tumor cells in a concentration‐dependent manner, suggesting the versatility of this synergetic strategy since GLUT1 protein is overexpressed in diverse tumors.^[^ ^19^ ^]^ The flow cytometry results show that the total (early and late) cell apoptotic rate of (Gen + Cur)@FOS (50 mg L^−1^) group is 63.9%, which is significantly higher than that of Cur@FOS (21.45%) and Gen@FOS (48.4%) group (Figure 4d), respectively. This is consistent with the corresponding CCK‐8 analysis result (Figure 4b), further confirming that Gen‐mediated tumor starvation promotes the apoptosis of tumor cells. These demonstrate that the inhibition of GLUT1 induced by Gen amplifies the sensitivity of tumor cells toward the chemotherapy drug of Cur, and thus the treatment effect is greatly enhanced by such “valve‐closing” starvation‐mediated stress sensitization.

**Figure 5:**
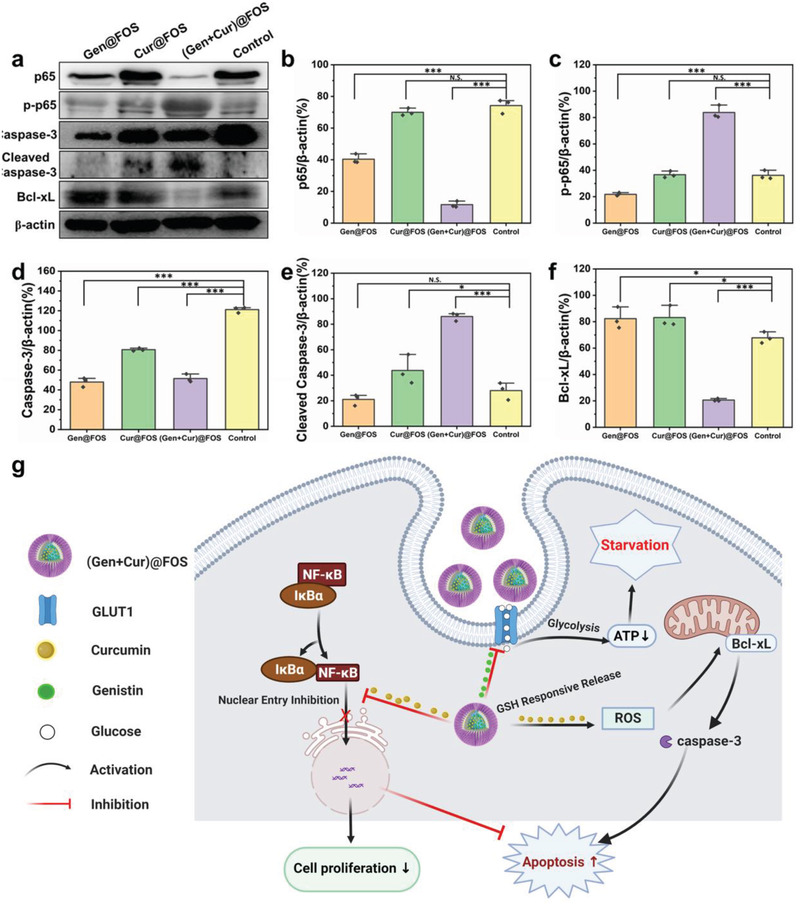
a) Effects of different treatments on the expression of p65, p‐p65, caspase‐3, cleaved caspase‐3, Bcl‐xL, and *β*‐actin in HeLa cells treated with (Gen+Cur)@FOS for 48 h. b–f) Quantification for the expression of corresponding proteins from Figure 4a. g) Schematic diagram of the apoptosis mechanism of (Gen + Cur)@FOS. *β*‐actin expression levels serve as the loading controls. Experiments were repeated three times with similar results. Statistical significances were calculated via Student's *t*‐test. *P < 0.05, **P < 0.01, and ***P < 0.001.

The collaborative mechanism of (Gen + Cur)@FOS to tumor cells was further investigated at the molecular level (**Figure** **5**). The expressions of some important protein members (NF‐*κ*B p65, caspase‐3, and Bcl‐xL) in the apoptosis signaling pathway were determined and quantified by immunoblotting analysis after treating HeLa cells with (Gen + Cur) @ FOS for 24 h (Figure S11, Supporting Information) and 48 h (Figure 5a–f). NF‐*κ*B p65 is reported as a key checkpoint to balance cells’ survival and death by regulating its phosphorylation degree (p‐p65) after entering the cell nucleus.^[^ ^20^ ^]^ It is observed that the (Gen + Cur)@FOS group presents the highest phosphorylation level of NF‐*κ*B (p65), compared to the control and single Gen/Cur@FOS groups (Figure 5b,c). It is known that caspase‐3 is the main end‐shedding enzyme in the apoptotic process, which will be autocleaved and activated when the apoptotic process is initiated.^[^ ^21^ ^]^ As shown in Figure 5a,e, the level of cleaved caspase‐3 is significantly increased by 3.2‐fold in (Gen + Cur)@FOS group, compared with the control group. On the contrary, the level of antiapoptotic protein Bcl‐xL in (Gen + Cur)@FOS group exhibits the most remarkable decrease (Figure 5f) among the four groups.^[^ ^22^ ^]^ In conclusion, the (Gen + Cur)@FOS‐treated HeLa cells present a much higher level of phosphorylated NF‐*κ*B (p‐p65) and cleaved‐caspase 3, as well as a much lower level of antiapoptotic protein Bcl‐xL, suggesting that the Gen‐induced starvation can effectively weaken the resistance of tumor cells to Cur‐induced apoptosis and promote the tumor cells’ apoptosis for the amplification of chemotherapeutic effect (Figure 5g).

**Figure 6:**
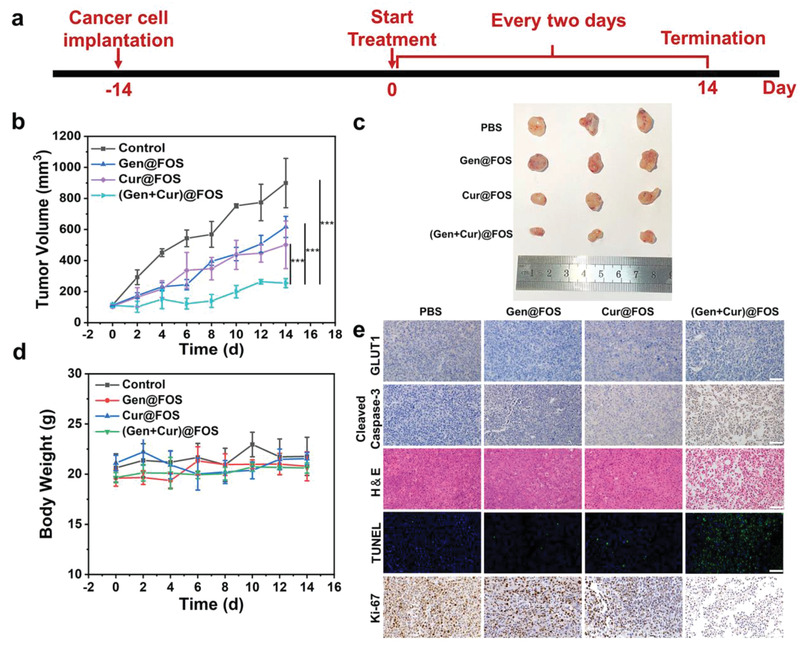
a) Overview of the (Gen + Cur)@FOS experimental design. b) Tumor volume changes of different treatment groups. c) Photos of tumor tissues from the HeLa mouse model after 14 days treatment. d) The body weight curves of mice in different groups. e) Immunohistochemical analyses of GLUT1, cleaved caspase‐3, Ki‐67, immunofluorescence analysis of TUNEL, and H&E staining of xenografted HeLa tumor sections after different treatments at day 14. Scale bar: 50 µm. Statistical significances were calculated via Student's *t*‐test. *P < 0.05, **P < 0.01, and ***P < 0.001.

The pharmacokinetics and tumor targeting ability of the (Gen+Cur)@FOS nanomedicine were investigated before in vivo antitumor therapy investigation. As shown in Figure S12 of the Supporting Information, the fluorescence signal of Nile Red@FOS in the blood of mice decreases with time increasing and its dependence on time fits well with a two‐compartment pharmacokinetic model. Based on the fitted curves, the in vivo circulating half‐life (*t*_1/2_) of Nile Red@FOS in blood stream was calculated to be 1.51 h, and the shifting time of Nile Red@FOS from the distribution phase to the elimination phase was determined as 0.49 h. The tumor targeting capability of FOS was tested by in vivo fluorescence imaging method, where a classic fluorescent label of zinc phthalocyanine (ZnPc) with broader emission was encapsulated into the FOS nanocarrier. As shown in Figure S13 of the Supporting Information, much stronger fluorescence intensity of ZnPc@FOS than that of free ZnPc is observed at the tumor site, indicating that the ZnPc@FOS can effectively target the tumor site owing to the EPR effect. To evaluate the in vivo therapeutic effects of (Gen + Cur)@FOS, the xenografted cervical cancer model was established by subcutaneous injection of HeLa cells into the right legs of Balb/c nude mice. When the tumor volume reached ≈100 mm^3^, HeLa tumor‐bearing mice were randomly divided into four groups treated with PBS, Gen@FOS, Cur@FOS, and (Gen + Cur)@FOS (equivalent Gen/Cur dose: 10 mg kg^−1^), respectively (**Figure** **6**a). The body weight and tumor volumes were recorded every two days during 14 days of the therapeutic period. As shown in Figure 6b, fast tumor growth is observed in the PBS control group, however, the tumor growth in Gen@FOS, Cur@FOS, or (Gen + Cur)@FOS group is inhibited in varying degrees. Especially, the mice treated with (Gen + Cur)@FOS displayed a most significantly reduced tumor growth rate. The digital photos of tumors excised from the mice further verify that the (Gen + Cur)@FOS is the most effective in inhibiting tumor growth among the four groups (Figure 6c). According to the variation of the tumor volume, the tumor inhibition rate of Gen@FOS, Cur@FOS, and (Gen + Cur)@FOS was calculated to be 31.42%, 44.27%, and 71.70%, respectively. The highest tumor inhibition rate of (Gen + Cur)@FOS demonstrates that the designed “valve‐closing” starvation strategy can greatly enhance the sensitivity of tumors to chemotherapy and amplify the chemotherapeutic efficacy. Moreover, there are no obvious fluctuations in the body weight of mice for each group during the treatment, as evidenced by Figure 6d. Importantly, (Gen + Cur)@FOS shows no visible adverse effects on normal tissues, as demonstrated by the hematoxylin and eosin (H&E) staining of major organs (heart, liver, spleen, lung, and kidney) of the mice (Figure S14, Supporting Information). Hematological parameters imply that some normal blood indicators were affected by Gen@FOS and Cur@FOS treatments, while no evident impacts of (Gen + Cur)@FOS treatment on the blood indicators were found (Figure S15, Supporting Information), confirming the high biosafety of this therapeutic approach in vivo.

Furthermore, the underlying mechanism for the enhanced therapeutic effects of (Gen + Cur)@FOS was investigated by immunohistochemistry. As shown in Figure 6e, the expression of GLUT1 at the tumor site is obviously inhibited by both the Gen‐containing nanomedicines, and the level of cleaved caspase‐3 is significantly increased by the Cur‐containing nanomedicines, especially the (Gen + Cur)@FOS group performed most obviously, which are consistent with the cell‐level results in Figure 5. This indicates that (Gen + Cur)@FOS can effectively inhibit GLUT1 expression (“valve‐closing”) to starve the tumors and render them more sensitive to Cur, further promoting the apoptotic effect through increased activation of caspase‐3 and thus achieving excellent tumor growth inhibition. Benefiting from such starvation‐mediated chemotherapy sensitization, (Gen + Cur)@FOS presents the highest level of cell apoptosis and necrosis, as evidenced by H&E staining results of tumor (Figure 6e). Moreover, terminal‐deoxynucleotidyl transferase‐mediated dUTP‐biotin nick end labeling (TUNEL) staining results also show that (Gen + Cur)@FOS leads to the most DNA breaks and apoptosis in HeLa tumors, which is in good agreement with its slowest tumor growth (Figure 6e). Besides, compared to the other treatment groups, the drastically reduced expression of Ki‐67 as cell proliferation index in (Gen + Cur)@FOS group indicates its significant inhibition of tumor cell proliferation. These results fully demonstrate that (Gen + Cur)@FOS can effectively inhibit tumor growth while minimizing damage to normal tissues by the nanomedicine‐enabled differential stress sensitization between cancer and normal cells.

## 3 Conclusion

In summary, we have demonstrated a novel “valve‐closing” starvation strategy to amplify the chemotherapy effects based on a stability‐controllable organosilica hybrid micelles nanomedicine ((Gen + Cur)@FOS), which is fabricated by encapsulating Gen as the GLUT1 inhibitor and Cur as the chemotherapeutic agent into FDA‐approved F127 micelles crosslinked with a disulfide bonds‐doped hybrid organosilica core. In this nanomedicine, Gen can effectively block the glucose uptake pathway for starving the HeLa tumors by inhibiting the GLUT1 expression (“valve‐closing”) and subsequently enhancing their sensitivity toward the Cur, thus resulting in significantly improved tumor therapeutic efficiency and reduced side effects in vivo. Such a “valve‐closing” starvation strategy for amplification of tumor‐specific chemotherapy provides a promising paradigm for high‐efficacy and low‐toxic cancer treatment.

## Conflict of Interest

The authors declare no conflict of interest.

## Supporting information

Supporting Information

## Data Availability

Research data are not shared.
