# Supplementary material for: A “Valve‐Closing” Starvation Strategy for Amplification of Tumor‐Specific Chemotherapy
Source: Adv Sci (Weinh). 2022 Jan 17;9(8):2104671. doi: 10.1002/advs.202104671 (PMC8922125; doi:10.1002/advs.202104671)
Supplement: Supplementary file 1 — Supporting Information [file ADVS-9-2104671-s001.pdf]

ADVANCED SCIENCE
Open Access

Supporting Information

for *Adv. Sci.,* DOI: 10.1002/advs.202104671

A "Valve-Closing" Starvation Strategy for Amplification of Tumor-Specific Chemotherapy

Xianglong Li, Cong Jiang, Qinghua Wang, Shaobo Yang, Yuanyuan Cao, Ji-Na Hao, Dechao Niu, Yan Chen, Bo Han, Xin Jia, Peng Zhang*, and Yongsheng Li**

Supporting Information

A "Valve-Closing" Starvation Strategy for Amplification of Tumor-Specific Chemotherapy

Xianglong Li, Cong Jiang, Qinghua Wang, Shaobo Yang, Yuanyuan Cao, Ji-Na Hao*, Dechao Niu, Yan Chen, Bo Han, Xin Jia, Peng Zhang*, and Yongsheng Li*

Experimental Section

Chemicals: Pluronic F127 ($PEO_{100}PPO_{65}PEO_{100}$, where PEO represents polyethyleneoxide and PPO represents polypropyleneoxide) and (3-mercaptopropyl)trimethoxysilane (MPTMS) were purchased from Sigma-Aldrich. Curcumin (Cur), and Glutathione (GSH) were purchased from TCI (Japan). Genistein (Gen) was purchased from Aladdin Reagent Co., Ltd (Shanghai). NH_4OH was purchased from Sinopharm Chemical Reagent Co., Ltd (Shanghai). All chemicals were used as received without further purifications.

Characterizations: The morphologies of samples were measured by JEM-2100 TEM (JEOL, Japan). The sizes and Zeta potentials of samples were measured by a Malvern Zetasizer (Nano ZS, Malvern, UK). UV-visible spectra of samples were measured by a UV-visible spectrophotometer (Shimadzu, UV-3600, Japan). The Fourier transform infrared (FT-IR) spectra of the samples were measured with Nicolet iS50 (Thermo Scientific, China). The Raman spectra of the samples were measured by the Laser Micro-Raman spectrometer (invia reflex, Renishaw, UK).

Synthesis of organosilica hybrid micelles (FOS) and (Gen+Cur)@FOS: FOS is synthesized by the classic thin-film hydration method. Briefly, F127 (600 mg) and ethanol (10 mL) were dissolved in a 250 mL round bottom flask, and the ethanol was evaporated at 45 ºC to obtain a micellar film. Subsequently, the micellar film was re-dissolved in the water (10 mL) by rotating at a low speed at 45 ºC to obtain the F127 micellar aqueous solution. Subsequently, MPTMS (300 µL) and NH_4OH (50 µL) were added into the F127 micellar aqueous solution.

After the solution was stirred at room temperature for 24 h, the FOS was collected and dialyzed using a membrane with a molecular weight cutoff (MWCO) of 8000-14000 in ultra-pure water to remove the impurities. The final FOS aqueous solution is stored at 4 °C for further use.

To synthesize Gen@FOS, Cur@FOS, and (Gen+Cur)@FOS, genistein (10 mg) and curcumin (10 mg) were separately or simultaneously dissolved in ethanol (10 mL) containing F127 (600 mg), and then proceed with the above process.

For the cell uptake experiment, Nile Red was chosen as the fluorescent dye to label FOS. The preparation of Nile red@FOS is the same as the above experimental procedure, in which F127 (600 mg) and Nile red (5 mg) were used.

In vitro drug release: To determine the GSH-responsive drug release performance of (Gen+Cur)@FOS, 1 mL of (Gen+Cur)@FOS with a known drug content (The mass ratio of Gen:Cur = 1:1) was placed in a dialysis bag with Mw=8000-14000, and then 40 mL of PBS (pH=7.4, 5% DMSO) with different GSH concentrations (0 mM, 2 mM, 10 mM) was added in the container, which was kept in dark to prevent the drug from decomposing. According to the predetermined time intervals, 1 mL of external buffer solution was taken out for UV absorption measurement, and meanwhile, 1 mL of fresh buffer solution was added to keep the release environment consistent.

Cell culture: The HeLa, A549, H1299, HCT116, LO2, and HaCaT cells were purchased from ATCC. These cells were cultured and maintained in normal Dulbecco's modified eagle medium (DMEM) containing 10% fetal bovine serum (FBS, Gibco, South America) and 1% penicillin/streptomycin. Cell cultures were maintained in an incubator at 37 °C in a humidified atmosphere with 5% CO_2.

Glucose uptake evaluation: HeLa cells were seeded in 12-well plates at an initial density of 5 $\times 10^5$ cells/well, and treated with culture medium containing Gen@FOS ([Gen]= 3.125, 6.25,

12.5, 25, and 50 mg/L) for 48 h. Then the glucose uptake capability in Gen@FOS-treated HeLa cells was measured by the Glucose Uptake Assay Kit-Red (Dojindo, UP03).

Intracellular glucose evaluation: HeLa cells were seeded in 12-well plates at an initial density of 5×10^5 cells/well, and treated with culture medium containing Gen@FOS ([Gen]= 3.125, 6.25, 12.5, 25, and 50 mg/L) for 48 h. Then the intracellular glucose contents of Gen@FOS-treated HeLa cells were measured by the Glucose Assay Kit-WST® (Dojindo, G264).

Intracellular lactate dehydrogenase (LDH) evaluation: HeLa cells were seeded in 12-well plates at an initial density of 5×10^5 cells/well, and treated with culture medium containing Gen@FOS ([Gen]= 3.125, 6.25, 12.5, 25, and 50 mg/L) for 48 h. Then the LDH Assay Kit-WST® (Dojindo) was used to measure the intracellular LDH contents of Gen@FOS-treated HeLa cells.

Intracellular adenosine triphosphate (ATP) evaluation: Hela cells were seeded in 12-well plates at an initial density of 5×10^5 cells/well, and treated with culture medium containing Gen@FOS ([Gen]=3.125, 6.25, 12.5, 25, and 50 mg/L) for 48 h. Then, the intracellular ATP content was measured by assay kit (Beyotime, S0027).

Western blot: HeLa cells were incubated in 12-well plates, and digested and collected after indicated treatments. The cells were lysed with EDTA-free cell lysate containing complete protease inhibitors and centrifuged at 10,000 g for 10 min, and then boiled with Laemmli sample buffer for 5 min. Subsequently, the cell lysate (20 mg protein) was separated on 12% SDS-PAGE, transferred to a PVDF membrane, and blocked with TBST containing 5% BSA (1:1000 dilution) before incubating with the primary antibody (overnight) and the secondary antibody (diluted 1:3000) at room temperature for 2 h. Finally, the ECL chemiluminescence kit (Beyotime, P0018FS) was used for western blot analysis. ImageJ software (NIH) was used for densitometric analysis, and the cell expression was standardized according to housekeeping protein.

Cell uptake: HeLa cells were incubated in 6-well plates for 24 h. After 24 h of adherence and stable growth, the HeLa cells were treated with new culture medium containing Nile red@FOS for different times. Finally, the cells were trypsinized, washed, and resuspended in 0.5 mL of PBS and detected by flow cytometry (BD Accuri C6, USA).

In vitro cytotoxicity assessments: The HeLa, A549, H1299, HCT116, LO2, and HaCaT cells were seeded into 96-well cell culture plates at an initial density of 5 $\times 10^3$ cells/well. After 24 h of adherence and stable growth, the cells were treated with culture medium containing different concentrations of samples (The ratio of Gen to Cur in (Gen+Cur)@FOS was 1:1). After 48 h of incubation, the culture mediums were discarded and the Cell Counting Kit-8 assay (CCK-8, Beyotime, C0037) was used to quantify cell viabilities.

HeLa cells were incubated in 6-well plates for 24 h. After the indicated treatments, cells were trypsinized, washed, and resuspended in 0.5 mL of binding solution, followed by the incubation with the Annexin V-FITC/PI Apoptosis Detection Kit (KeyGEN BioTECH, KGA108) in dark for 15 min. Then, the HeLa cells were rinsed with PBS twice and analyzed immediately using a flow cytometer (BD Accuri C6, USA).

In vivo pharmacokinetic study: Nile Red @FOS was injected into three healthy Kunming mice through the tail vein, and 10 μL of blood was removed by scoring the tail at 2, 8, 15, 30 min and 1, 2, 4, 8, 24 h. Then the fluorescence intensities at 630 nm were measured after the quired blood dispersion in saline containing sodium heparin (50 U/mL). The *in vivo* circulating half-life ($t_{1/2}$) of Nile Red @FOS was calculated by fitting a biexponential pharmacokinetic model, and its metabolic rate was calculated by fitting the slope of the curve.

In vivo therapy: Animal experiments were executed according to the protocol approved by the Laboratory Animal Management Committee of East China University of Science and Technology (approval number: ECUST-2020-04001). Female Balb/c nude mice (4-week-old, n = 12) were purchased from Zhejiang Weitong Lihua Laboratory Animal Technology Co.,

Ltd. 2×10^7 HeLa cells suspension was subcutaneously injected into the back of the hind leg. HeLa tumor xenografted nude mice were randomly divided into four groups of three mice each when tumors reached 100 mm^3, and intravenously injected PBS, Gen@FOS, Cur@FOS, and (Gen+Cur)@FOS every two days (Gen and Cur of all drug-containing groups are 10 mg/kg) by the tail, respectively. The body weight, tumor volume of Balb/c nude mice were measured and recorded every two days. The calculation formula for tumor volume is V (mm^3) $=1/2\times$length (mm)$\times$width^2 (mm). After 14 days, the mice were sacrificed and the tumors and main organs were removed for histopathological section analysis, immunofluorescence, and IHC analysis.

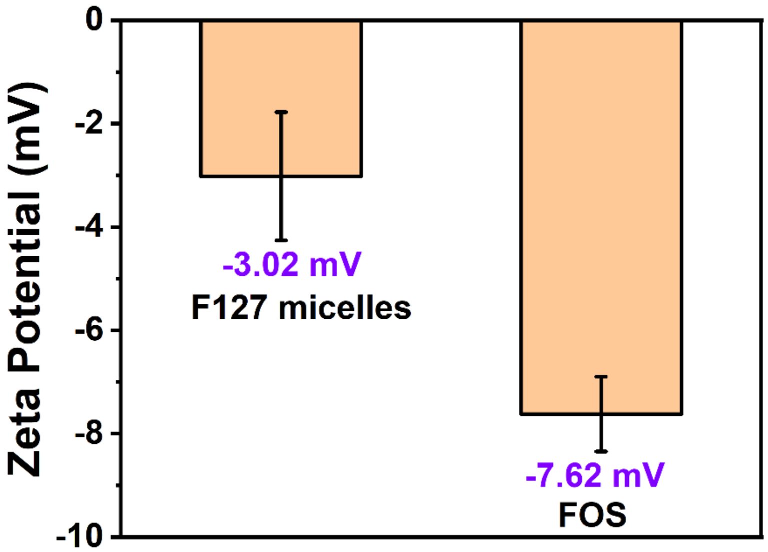

Figure S1. Zeta potentials of the F127 micelles and FOS.

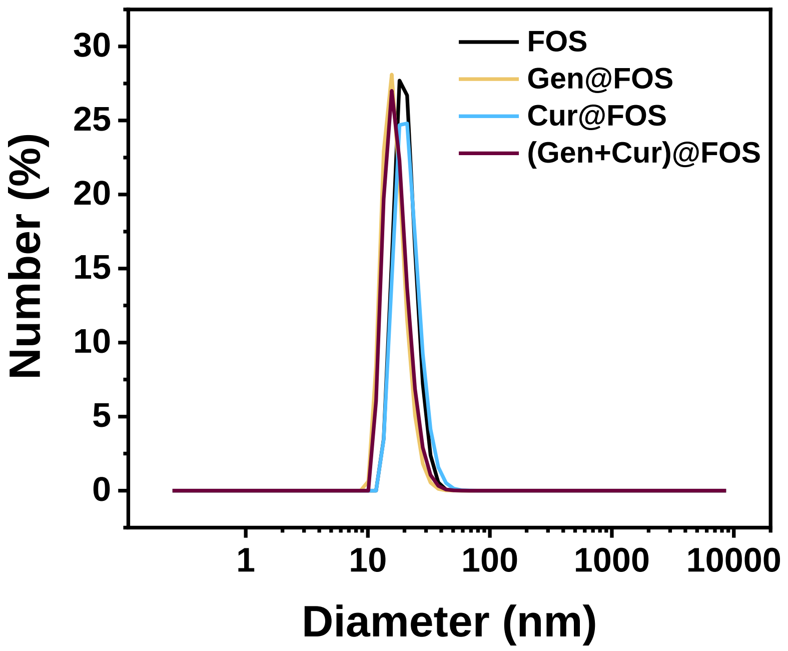

Figure S2. Hydrated size histogram of different materials by DLS measurement.

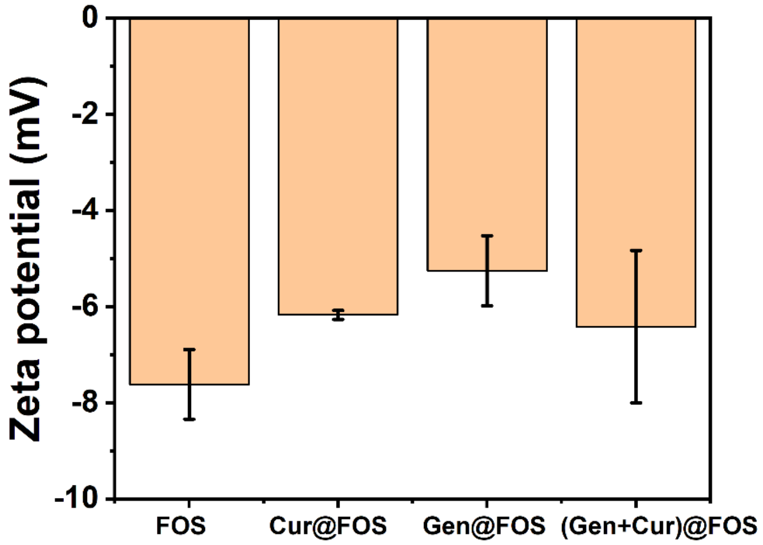

Figure S3. Zeta potential of different materials.

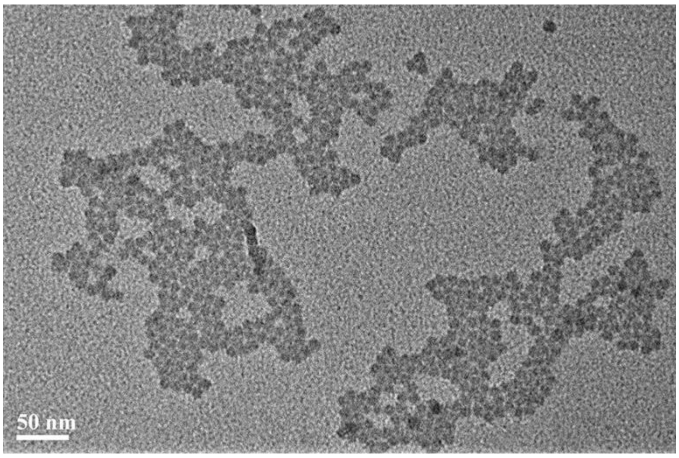

Figure S4. TEM image of Gen@FOS.

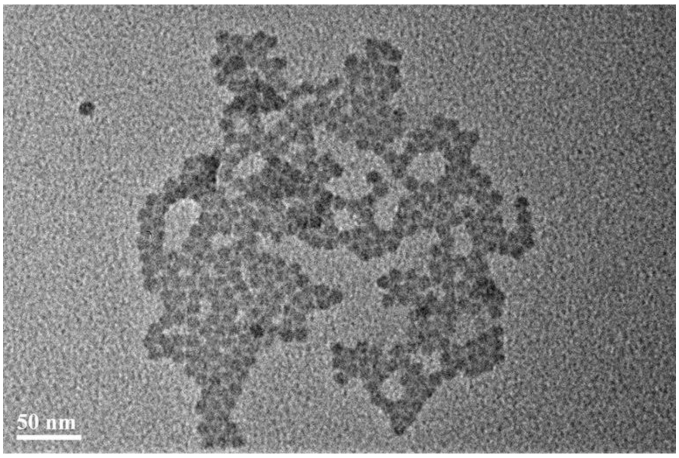

Figure S5. TEM image of Cur@FOS.

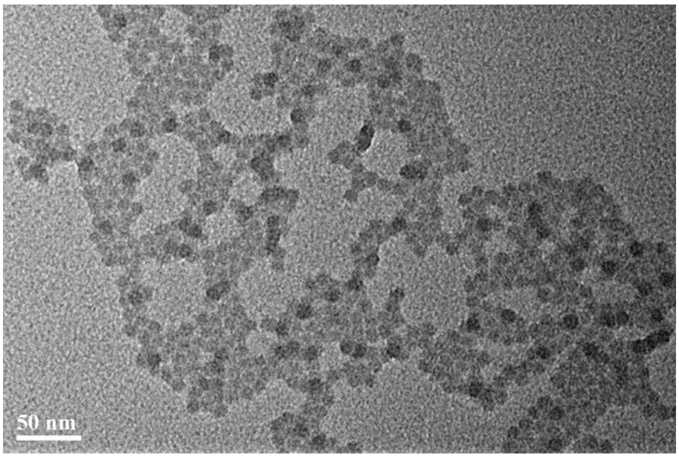

Figure S6. TEM image of (Gen+Cur)@FOS.

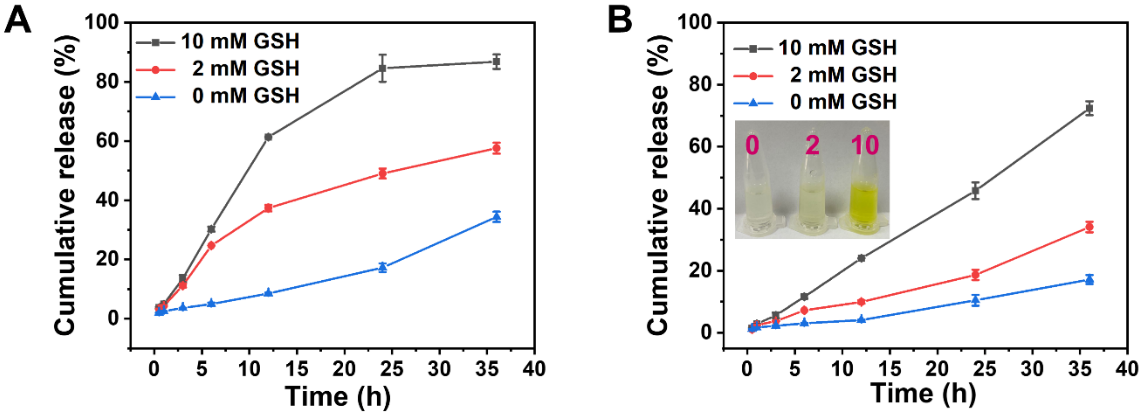

Figure S7. (A) Cumulative release curve of Gen from (Gen+Cur)@FOS at different GSH concentrations; (B) Cumulative release curve of Cur from (Gen+Cur)@FOS at different GSH concentrations, insert: digital photo of the external simulated release of the environmental solution.

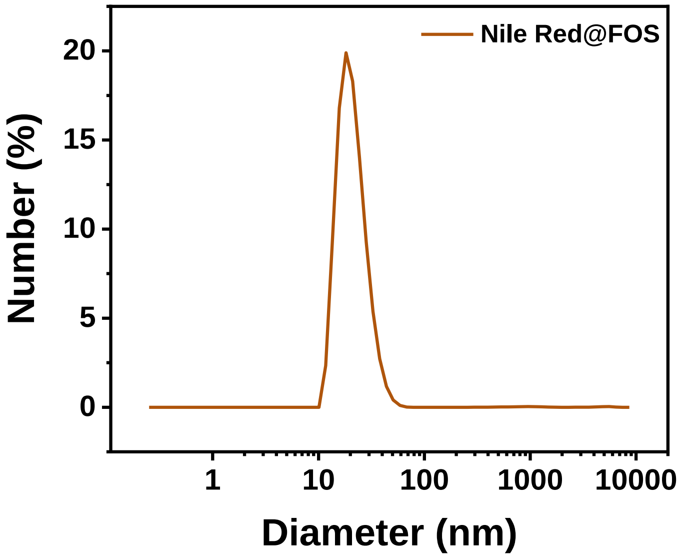

Figure S8. Particle size distributions of Nile Red@FOS determined by DLS in water.

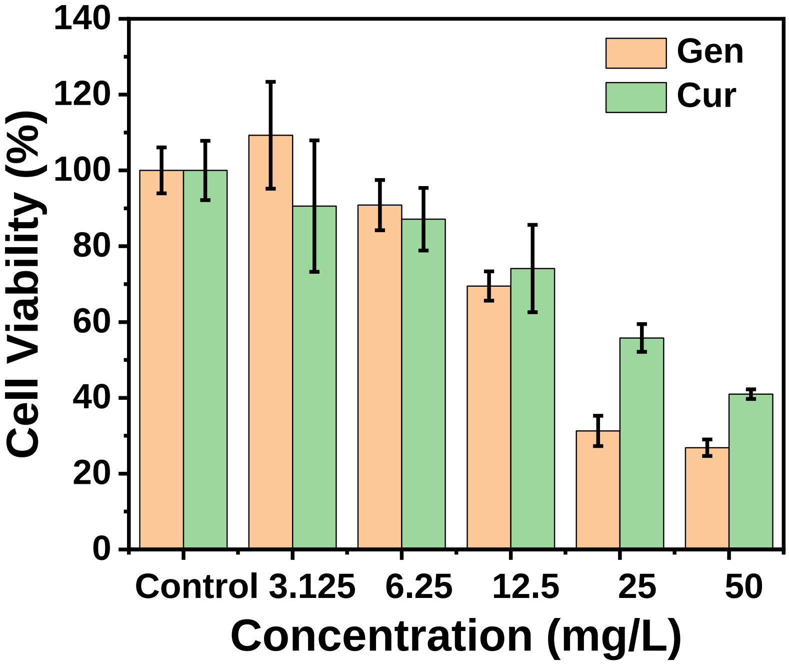

Figure S9. Cell viabilities of HeLa cells treated by Gen and Cur for 24 h.

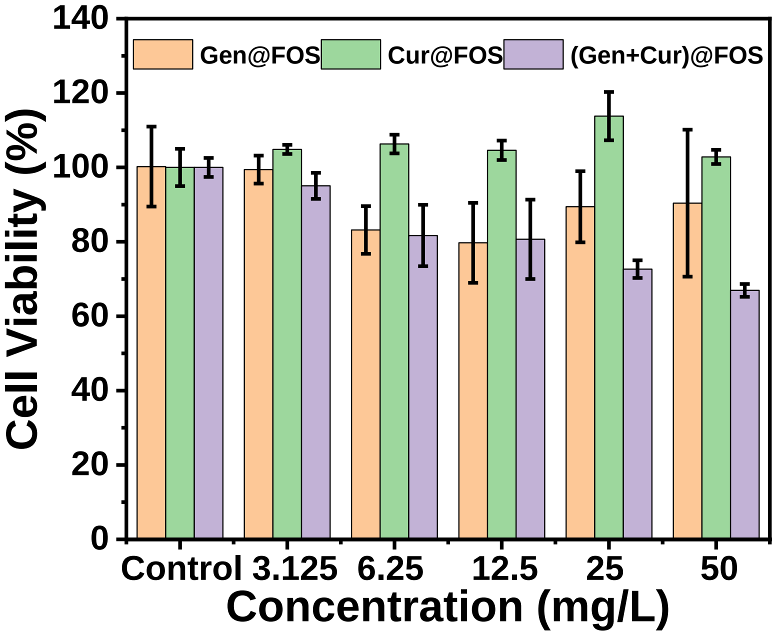

Figure S10. Cell viabilities of HeLa cells treated by Gen@FOS, Cur@FOS, and (Gen+Cur)@FOS for 24 h.

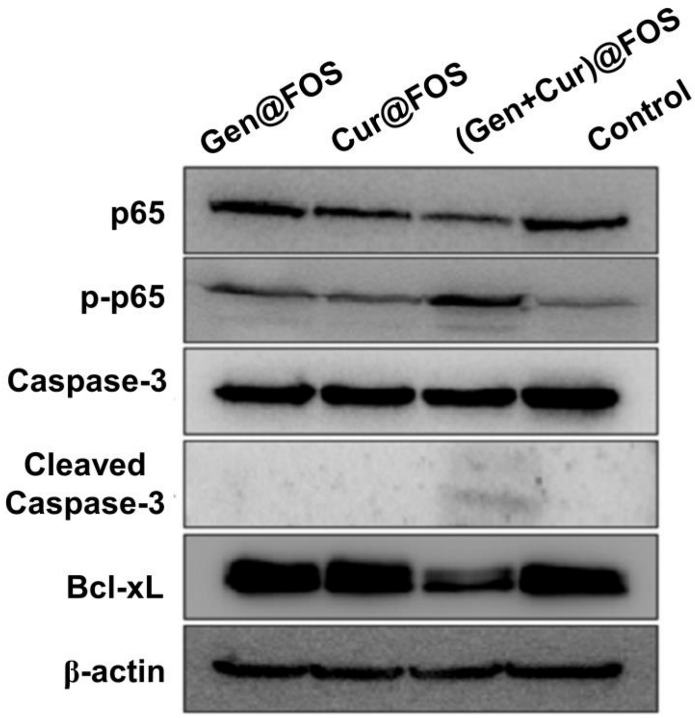

Figure S11. The expression of p65, p-p65, caspase-3, cleaved caspase-3, Bcl-xL, and β-actin in HeLa cells treated with Gen@FOS, Cur@FOS, (Gen+Cur)@FOS for 24 h, respectively.

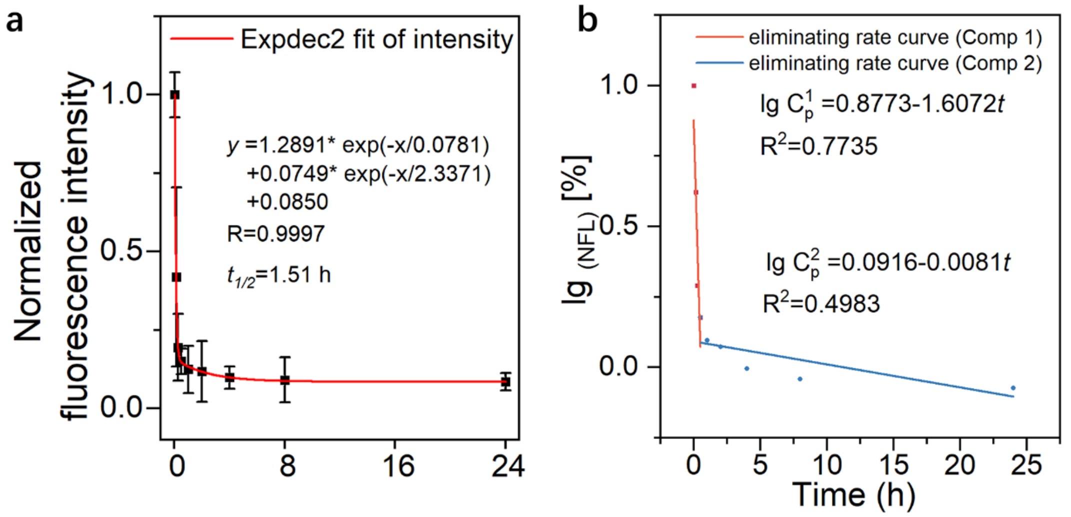

Figure S12. (a) The blood circulation curve of intravenously injected Nile Red@FOS by calculating the fluorescence intensity in the blood. (b) The eliminating rate curves of intravenously injected Nile Red@FOS from the blood circulation curve.

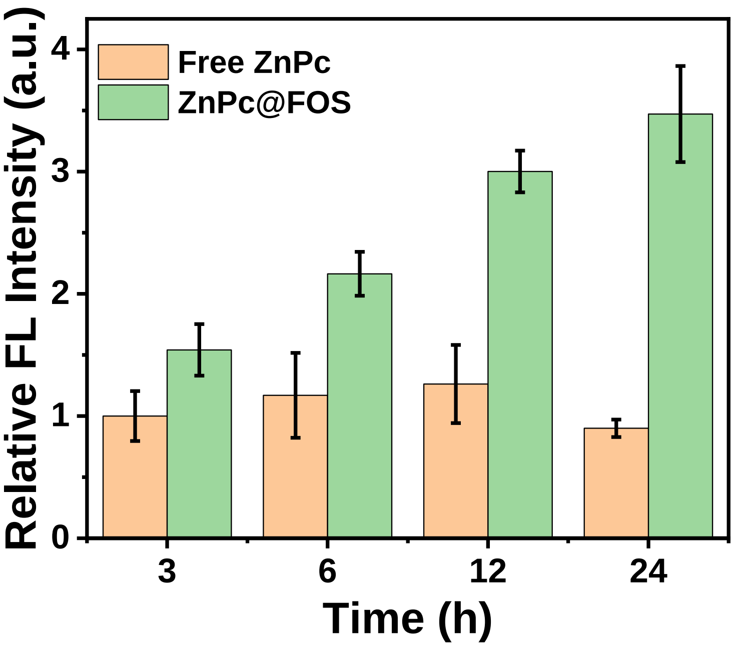

Figure S13. The fluorescence (FL) intensities of tumors from HeLa tumor-bearing mice after tail vein injection of free ZnPc and ZnPc@FOS (ZnPc dose: 60 μg) at different time.

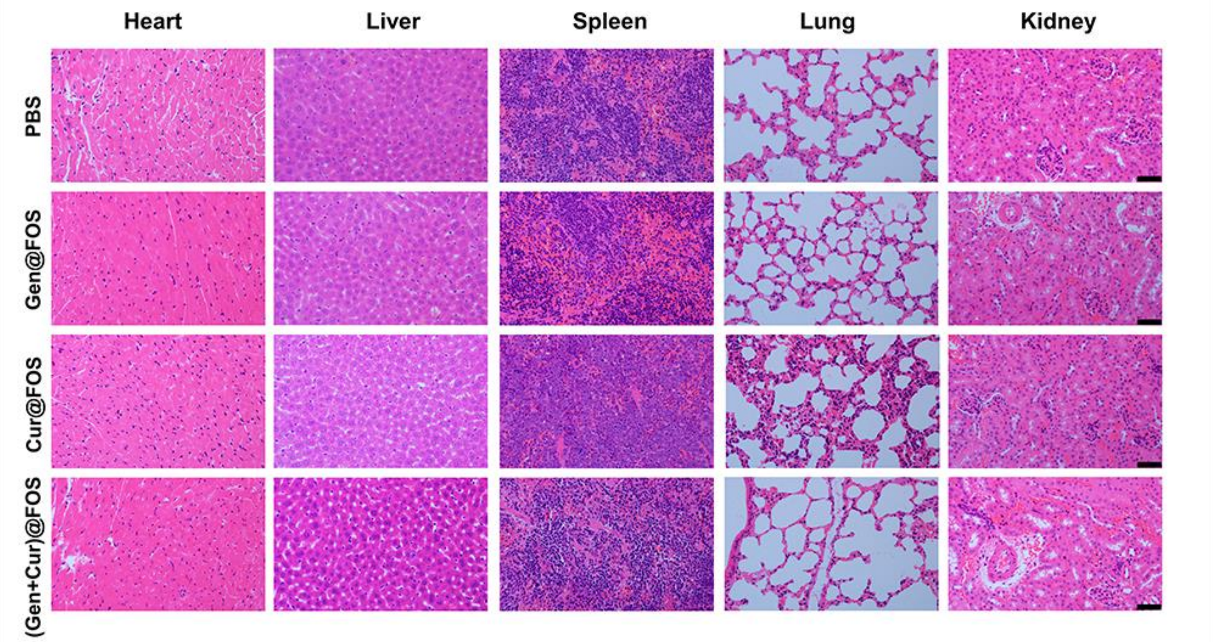

Figure S14. H&E staining of major organs harvested from HeLa tumor-bearing mice after different treatments at day 14. Each observation was performed on five parallel samples collected from different mice treated in one specific condition, and representative images are presented. Scale bars: 50 μm.

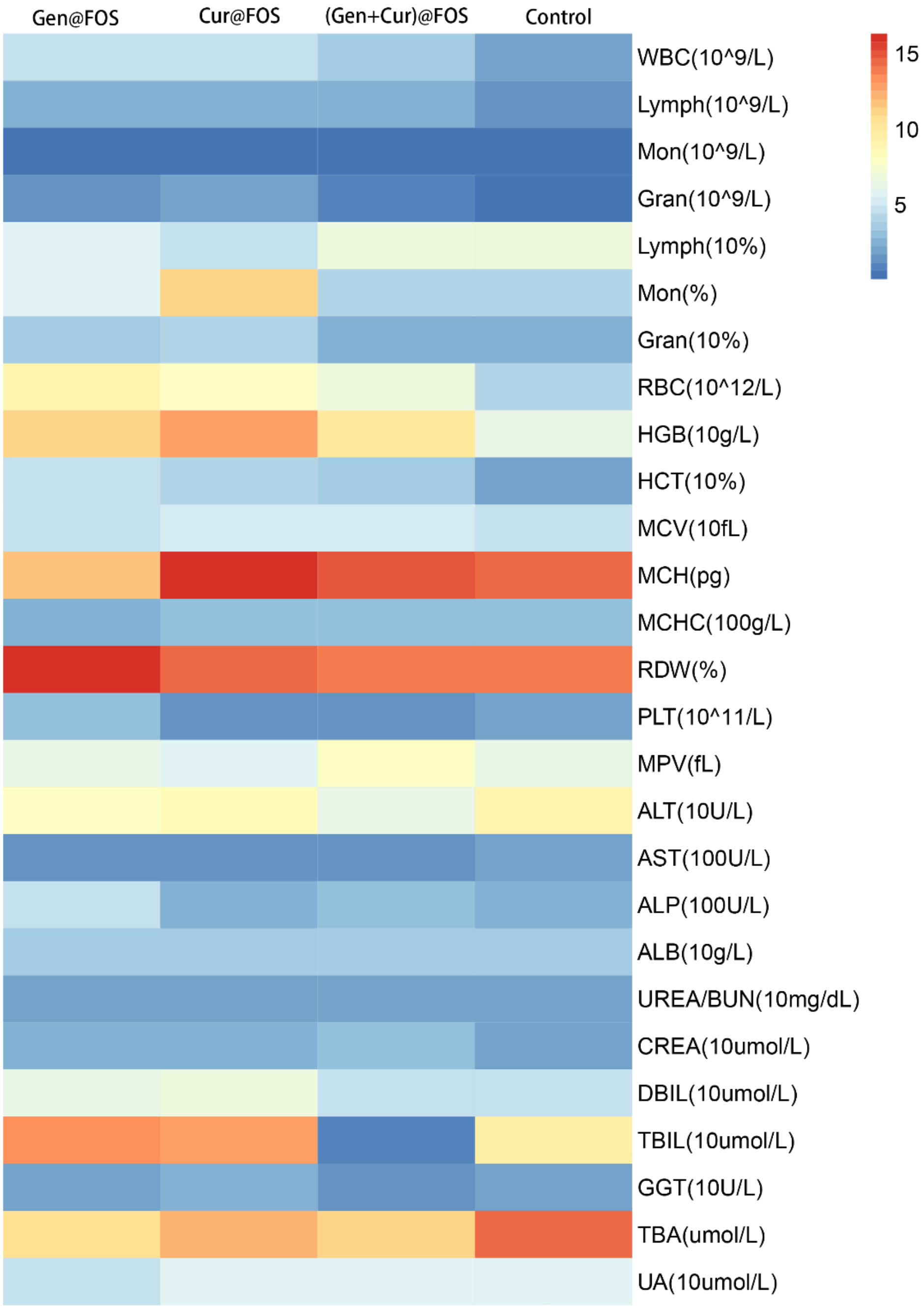

Figure S15. Hematological parameters of HeLa tumor-bearing mice after various treatments at day 14.
